# Mechanochemical synthesis of graphene oxide-supported transition metal catalysts for the oxidation of isoeugenol to vanillin

**DOI:** 10.3762/bjoc.13.141

**Published:** 2017-07-21

**Authors:** Ana Franco, Sudipta De, Alina M Balu, Araceli Garcia, Rafael Luque

**Affiliations:** 1 Departamento de Química Orgánica, Universidad de Cordoba Campus de Rabanales, Edificio Marie Curie (C-3), Ctra Nnal IV-A, Km 396, E14014, Cordoba, Spain; 2 Department of Chemical and Biomolecular Engineering, National University of Singapore, 4 Engineering Drive 4, 117585, Singapore

**Keywords:** H_2_O_2_, isoeugenol, mechanochemical synthesis, non-enzymatic process, vanillin

## Abstract

Vanillin is one of the most commonly used natural products, which can also be produced from lignin-derived feedstocks. The chemical synthesis of vanillin is well-established in large-scale production from petrochemical-based starting materials. To overcome this problem, lignin-derived monomers (such as eugenol, isoeugenol, ferulic acid etc.) have been effectively used in the past few years. However, selective and efficient production of vanillin from these feedstocks still remains an issue to replace the existing process. In this work, new transition metal-based catalysts were proposed to investigate their efficiency in vanillin production. Reduced graphene oxide supported Fe and Co catalysts showed high conversion of isoeugenol under mild reaction conditions using H_2_O_2_ as oxidizing agent. Fe catalysts were more selective as compared to Co catalysts, providing a 63% vanillin selectivity at 61% conversion in 2 h. The mechanochemical process was demonstrated as an effective approach to prepare supported metal catalysts that exhibited high activity for the production of vanillin from isoeugenol.

## Introduction

Vanillin is the main flavor and aroma compound in vanilla. It is an aromatic compound (4-hydroxy-3-methoxybenzaldehyde) containing two reactive functional groups that are useful for the synthesis of thermoplastic polymers [1–4].

Vanillin is one of the most important chemicals in the aroma industry, because it is abundantly used in food, pharmaceutical, cosmetic, and fine chemical industries. Therefore much attention has been paid to research on the improvement of its production [5].

At the present time only 1% of total vanilla production is from extraction of natural material. This extraction is a very long and expensive process [6]. The remaining 99% is being produced via chemical and biochemical routes. Biotechnology-based approaches, particularly enzymatic processes, have been well known for many years for vanillin production and are considerably less harmful to the environment. However, they have inherent disadvantages including comparatively high costs, slowness, difficult purification and the requirement of selected strains of microorganisms [7–9]. Major quantities (85%) of the world supply are still produced from petroleum-based intermediates, especially guaiacol and glyoxylic acid using the most employed Riedel process [10–11]. The classical synthetic routes are not “environment friendly” and the vanillin produced by these methods is considered to be of lower quality because it does not contain some trace components that contribute to the natural vanilla flavor.

Nowadays, 15% of the overall vanillin production comes from lignin, more precisely from lignosulfonates. Different products can be synthesized by lignin oxidation being vanillin the most well and valuable product. Recently, eugenol, isoeugenol and ferulic acid have been used as substrates for vanillin manufacturing due to their economic and commercial availability. These compounds are easily derived from lignin and have the common structural unit with that of vanillin, being potentially useful for vanillin production via simple oxidation pathways [12–14]. Photocatalytic oxidation has been reported for the production of vanillin where TiO_2_-based materials have been used as effective catalysts in recent years [15–18]. Although the conversion was high in some cases, vanillin selectivity was never significant. Another problem related to the slow reaction rates, unsuitable for commercial production. As a result, chemical oxidation pathways were also followed. To achieve faster kinetics and better selectivity of vanillin, homogeneous catalysts based on different transition metal salts/complexes were employed [14,19–21]. However, the selectivity of vanillin still remains an important issue.

In this work, we report the mechanochemical design of transition-metal-based catalysts supported on reduced graphene oxide support for the oxidation of isoeugenol into vanillin using H_2_O_2_ as oxidant. The catalytic support, RGO, a graphene derived material are normally produced by chemical reduction of graphene oxide (GO) [22–23].

The materials were prepared using a simple and effective ball milling approach and were characterized by different techniques.

## Results and Discussion

**Figure 1:**
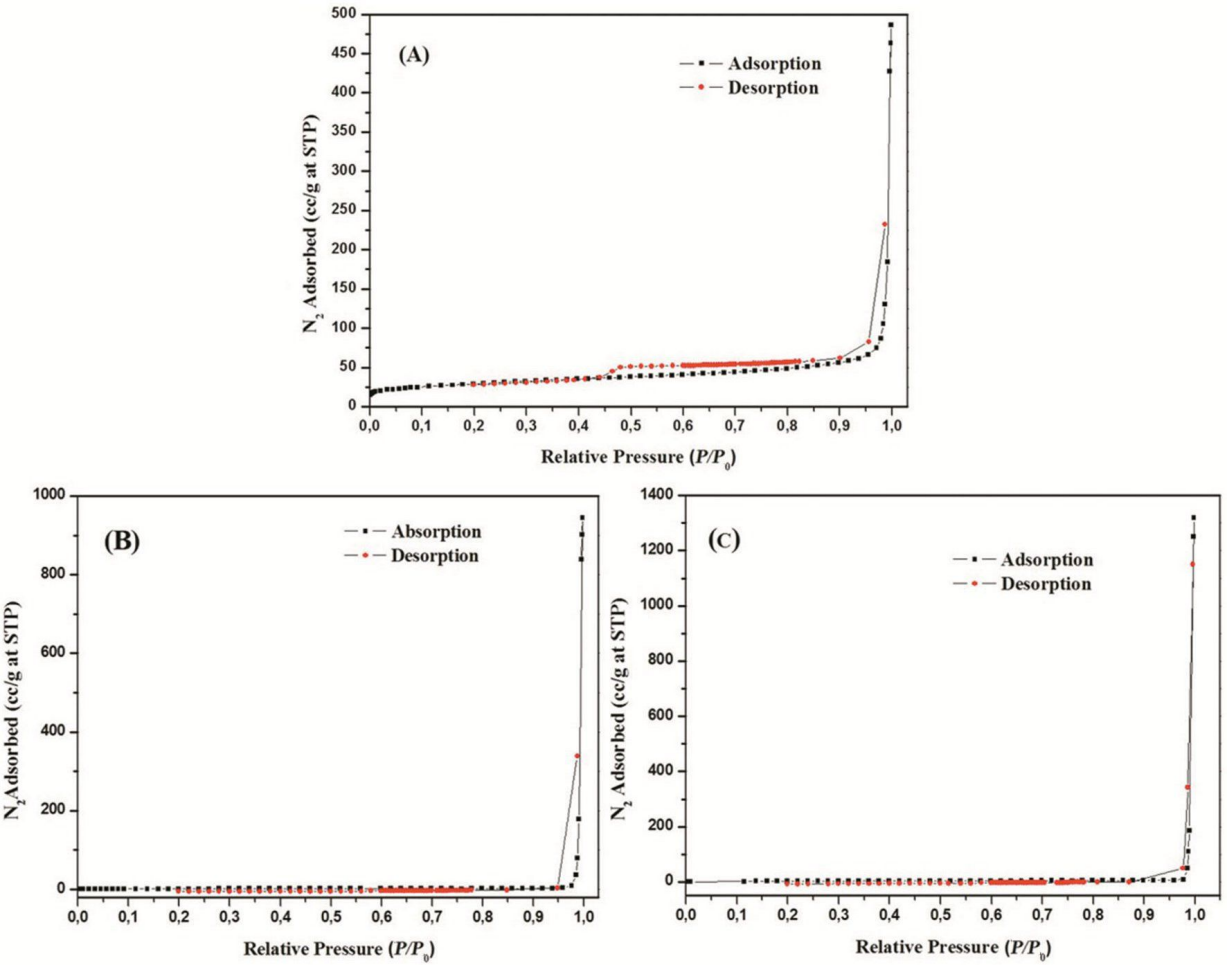
N_2_ isotherms of (a) RGO, (b) Fe/RGO, and (c) Co/RGO.

**Figure 2:**
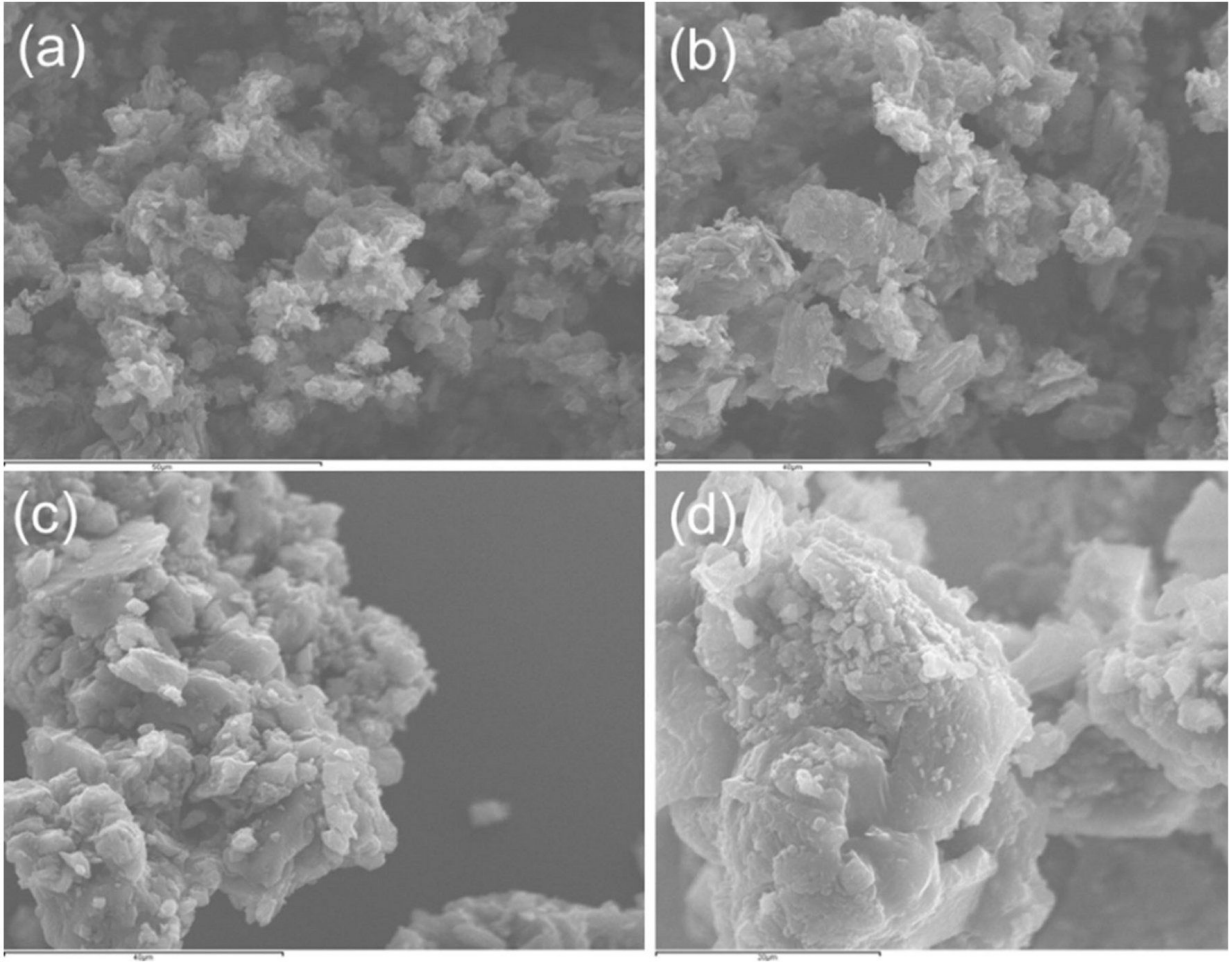
SEM images of (a and b) RGO, (c) 1% Fe/RGO, and (d) 1% Co/RGO.

The supported RGO materials were characterized by using several techniques including BET, SEM, TEM, XRD, and IR spectroscopy. N_2_ adsorption/desorption isotherms of the reduced graphene sample (Figure 1a) can be classified as type IV corresponding to the mesoporous materials. The RGO sample showed a BET surface area of 103 m^2^ g^−1^ with a pore diameter of 39 nm and pore volume of 0.74 cm^3^ g^−1^ (Table 1). After the ball milling with metal precursors, the mesoporous structure of RGO was found to be partially collapsed as observed from BET isotherms in Figure 1b and c. BET surface areas of metal supported RGO materials consequently decreased, with increased pore diameter and pore volume as a consequence of the structure deterioration observed after milling. Additional macroporosity (interparticular) was created upon milling, which increased both pore diameter and volume. SEM results also support the observation from BET analysis. The mesoporous nature of the RGO can be easily observed from SEM images (Figure 2a and b), whereas metal-supported RGO materials show a smooth surface with decreased crystallinity.

**Table 1:** Textural properties of RGO and NPs supported RGO materials.

Material	*S*_BET_^a^ (m^2^ g^−1^)	*D*_BJH_^b^ (nm)	*V*_BJH_^c^ (cm^3^ g^−1^)

RGO	103	39	0.74
1% Fe/RGO	<10	205	1.46
1% Co/RGO	<15	190	2.04

^a^*S*_BET_: specific surface area was calculated by the Brunauer–Emmet–Teller (BET) equation. ^b^D_BJH_: mean pore size diameter was calculated by the Barret–Joyner–Halenda (BJH) equation. ^c^V_BJH_: pore volumes were calculated by the Barret–Joyner–Halenda (BJH) equation.

**Figure 3:**
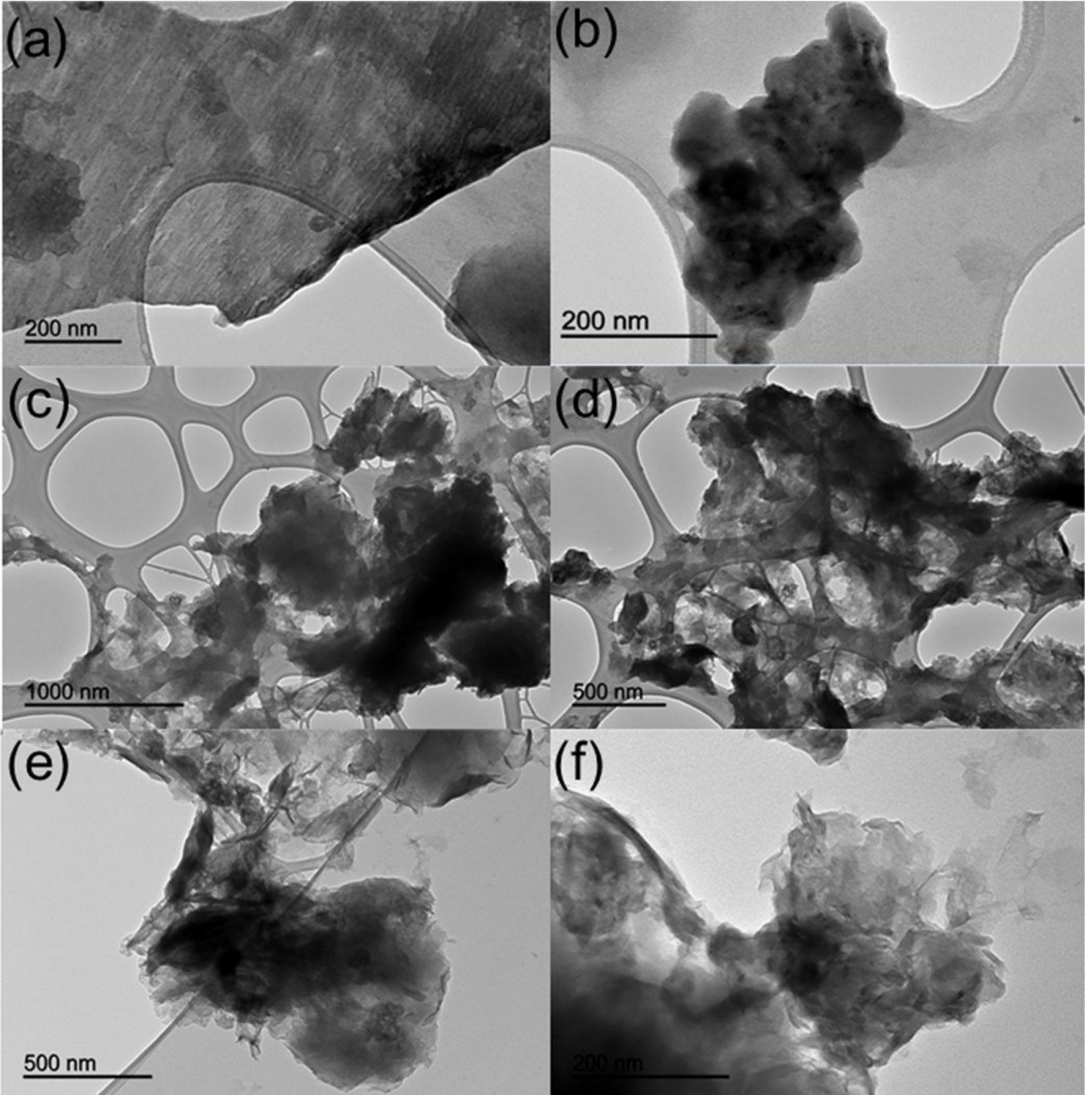
TEM micrographs at different magnifications of (a and b) RGO, (c and d) 1% Fe/RGO, and (e and f) 1% Co/RGO.

TEM images of RGO materials with different thickness show a sheet like morphology with different transparencies (Figure 3). Dark areas result from the superposition of several graphene oxide and/or graphene layers containing oxygen functional groups. Most transparent areas are from thinner films composed of a few layers of reduced graphene oxide from stacking nanostructure exfoliation. A significant collapse of the structure could be observed upon metal incorporation (see Figure 3, images c and d), although several domains remained to be almost unchanged as compared to those of RGO (see Figure 3f).

**Figure 4:**
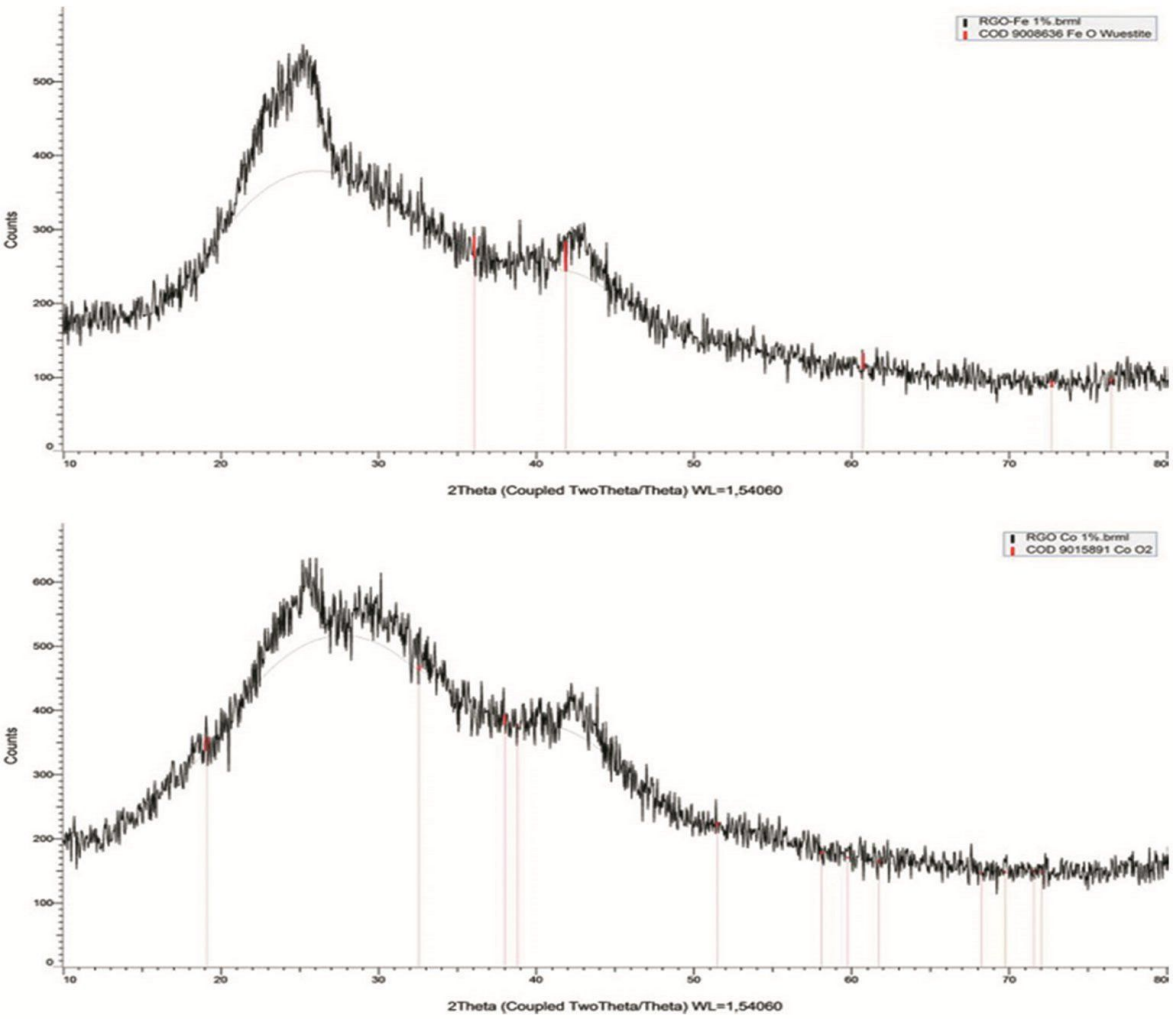
Powder XRD patterns of RGO supported Fe and Co NPs.

X-ray diffraction patterns of RGO-supported materials are shown in Figure 4. Two characteristic peaks at 2θ = 26° and 2θ = 43° correspond to the typical RGO material. The broad nature of the peak confirms the highly amorphous nature of the RGO support. A closer look at the figures pointed out the presence of iron in the form of a FeO/Fe_2_O_3_ mixture (mixed phases) as compared to a more pure CoO phase in the case of Co. Due to the amorphous nature of RGO and low metal loading, the corresponding metal oxide peaks could not be well resolved.

**Figure 5:**
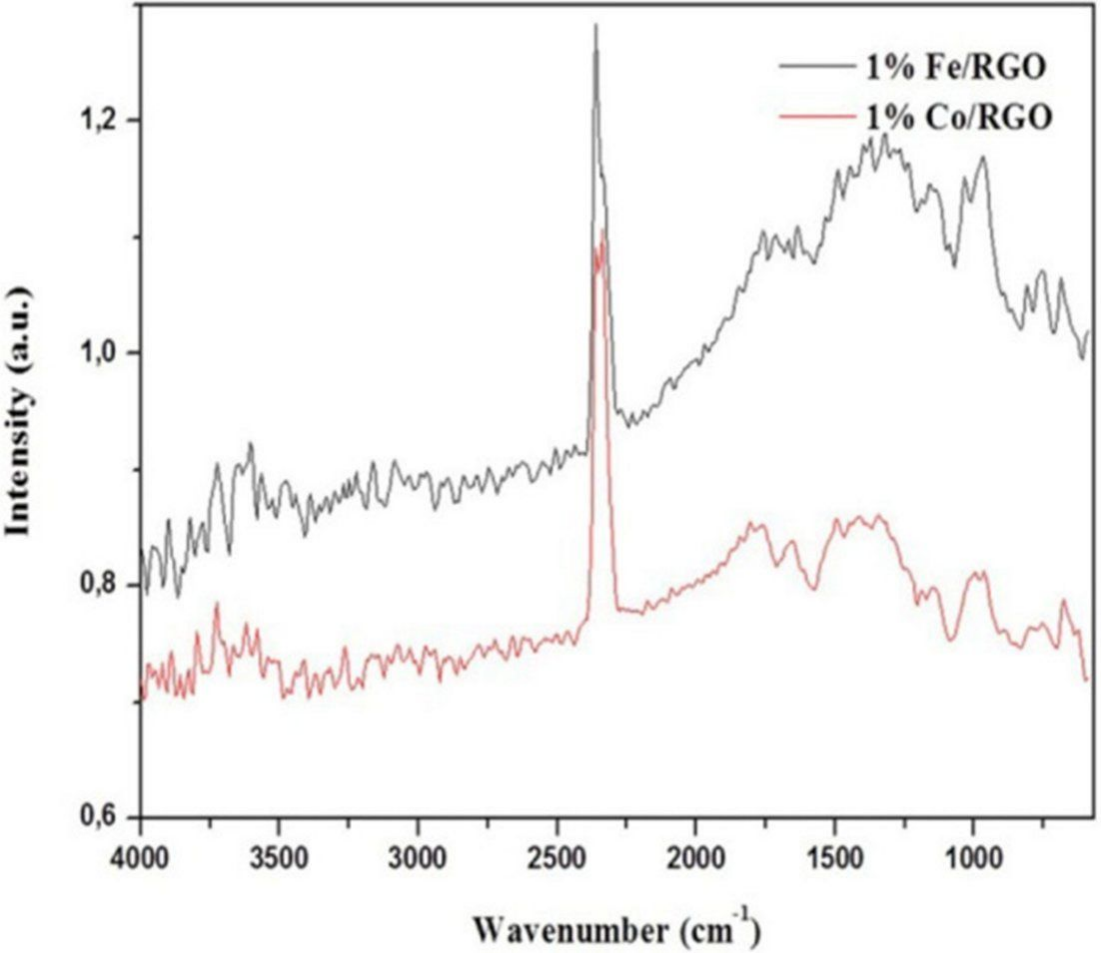
IR spectra of 1% Fe/RGO and 1% Co/RGO catalysts collected by using diffuse reflectance infrared transform spectroscopy (DRIFT) at room temperature.

Additionally, IR spectra (Figure 5) showed that there is no such peak in the range of 1700–1740 cm^−1^, indicating the absence of any oxidized groups such as carbonyl or carboxylic acid groups. One peak at around 1600 cm^−1^ could be observed that corresponds to C=C from aromatic groups.

Table 2 summarizes the experimental results for the oxidation of isoeugenol using supported RGO catalysts. Reaction conditions were optimized under various conditions. Blank runs (in absence of catalysts) were also performed, with a low conversion in the systems, which could be attributed to the effect of the strong oxidizing agent H_2_O_2_. However, the reaction produced a higher amount of ether compounds with a very low selectivity to vanillin. When RGO was used as catalyst, the conversion increased but the selectivity of vanillin was still lower than other side products. Importantly, metal incorporation on RGO support significantly increased both conversion and vanillin selectivity in the systems (Table 2, entries 3 and 4).

**Table 2:** Results for the catalytic oxidation of isoeugenol.^a^

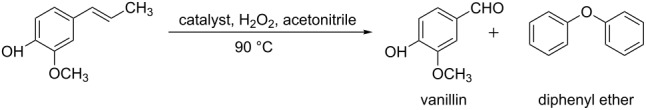						

Entry	Catalyst	Time (h)	Conversion (mol %)	Selectivity (mol %)		
	
				Vanillin	Diphenyl ether	Others

1.	blank	2	18	7	84	9
2.	RGO	2	39	26	47	27
3.	1% Fe/RGO	2	61	63	8	29
4.	1% Co/RGO	2	60	32	9	59
5.	blank	3	19	8	79	13
6.	RGO	3	41	25	47	28
7.	1% Fe/RGO	3	64	58	13	29
8.	1% Co/RGO	3	70	27	6	67
9.	blank	5	20	11	73	16
10.	RGO	5	54	19	30	51
11.	1% Fe/RGO	5	64	54	13	33
12.	1% Co/RGO	5	75	21	4	75
13.	blank	7	22	16	70	14
14.	RGO	7	59	26	23	51
15.	1% Fe/RGO	7	62	52	14	34
16.	1% Co/RGO	7	81	19	2	79

^a^Reaction conditions: 5 mmol isoeugenol, 1.2 mL H_2_O_2_, 8 mL acetonitrile, 0.1 g catalyst, 90 °C.

The optimum results were obtained after 2 h of reaction as seen in results from Table 2. The Fe-containing catalysts were found to be more selective than the Co-containing catalysts at similar conversions under otherwise identical reaction conditions. After prolonged reaction times, Fe/RGO remained selective towards vanillin, but Co/RGO experienced a significant drop in selectivity (although the conversion increased). This could be explained by the strong oxidizing nature of Co that might facilitate further reactions of vanillin to other compounds. To investigate the stability of the Fe/RGO and Co/RGO the materials were subjected to different reuses. The results showed a significantly decrease in the catalytic activity due to material deactivation.

## Conclusion

A simple mechanochemical ball milling process was used to prepare highly active transition-metal-supported reduced graphene oxide catalysts. The catalysts were used to produce the highly useful aromatic compound vanillin, by oxidizing naturally abundant isoeugenol. The catalysts showed good activity and vanillin selectivity at mild reaction conditions using H_2_O_2_ as oxidizing agent. A better selectivity was observed for the Fe-based catalyst.

## Materials and Methods

### Preparation of materials

In a typical synthesis of ball-milled materials, reduced graphene oxide (RGO) support, together with an appropriate amount of the iron precursor (FeCl_2_∙4H_2_O) to reach a theoretical 1% iron loading, was ground by using a Retsch-PM-100 planetary ball mill with a 25 mL reaction chamber and 8 mm stainless steel ball. Milling was conducted at 350 rpm for 10 min. The same protocol was used to design a 1% Co catalyst using the Co precursor Co(NO_3_)_2_∙6H_2_O. Graphene oxide was kindly donated by Nano Innova Technologies SL (http://www.nanoinnova.com).

### Characterization of materials

Materials were characterized by using N_2_ physisorption, powder X-ray diffraction (XRD), transmission electron microscopy (TEM), scanning electron microscopy (SEM) and diffuse reflectance infrared Fourier transform spectroscopy (DRIFT). N_2_ adsorption measurements were performed at 77 K by using a Micromeritics ASAP 2000 volumetric adsorption analyzer. The samples were degassed for 24 h at 30 °C under vacuum (*P*_0_ < 10^−2^ Pa) and subsequently analyzed. Surface areas were calculated according to the BET (Brunauer–Emmet–Teller) equation. Mean pore size diameter and pore volumes were measured from porosimetry data by using the BJH (Barret–Joyner–Halenda) method. Wide-angle X-ray diffraction experiments were performed on a Pan-Analytic/Philips X`pert MRD diffractometer (40 kV, 30 mA) with Cu Kα (λ = 0.15418) radiation. Scans were performed over a 2θ range between 10–80° at step size of 0.0188 with a counting time per step of 5 s. TEM images of the samples were recorded on JEM 2010F (JEOL) and Phillips Analytical FEI Tecnai 30 microscopes. SEM micrographs were recorded on a JEOL-SEM JSM-6610 LV scanning electron microscope in backscattered electron model at 3/15 kV. DRIFT spectra were recorded on a PIKE Technologies MB 3000 ABB at room temperature.

### Catalytic activity tests

In a typical experiment, isoeugenol (5 mmol) and 0.1 g catalyst, H_2_O_2_ (1.2 mL) and acetonitrile (8 mL) were heated at 90 °C under continuous stirring in a carrusel place reaction station. Products were analyzed at different time interval by GC Aligent 7890 fitted with a capillary column Petrocol 100 m × 0.25 nm × 0.5 μm and a flame ionization detector (FID). The results were finally confirmed by GC–MS.
